# Differential Expression Profiles and Bioinformatics Analysis of tRNA-Derived Small RNAs in Muscle-Invasive Bladder Cancer in a Chinese Population

**DOI:** 10.3390/genes13040601

**Published:** 2022-03-28

**Authors:** Chuan Qin, Zheng-Hao Chen, Rui Cao, Ming-Jun Shi, Ye Tian

**Affiliations:** Department of Urology, Beijing Friendship Hospital, Capital Medical University, Beijing 100068, China; qinchuanlove@126.com (C.Q.); chenzhmed@163.com (Z.-H.C.); caorui@whu.edu.cn (R.C.); shimingjun1127@126.com (M.-J.S.)

**Keywords:** tRNA-derived small RNA, muscle-invasive bladder cancer, sequencing, bioinformatics

## Abstract

Muscle-invasive bladder cancer (MIBC) leads to a large societal burden. Recently, tRNA-derived small RNAs (tsRNAs), a novel type of noncoding RNA (ncRNAs), have been identified. However, the expression patterns and functions of tsRNAs in MIBC have not yet been identified. Here, RNA sequencing, bioinformatics, and quantitative reverse transcription- polymerase chain reaction (qRT-PCR) were used to screen the expression profiles and predict the potential roles of tsRNAs in MIBC. Of 406 tsRNAs differentially expressed in MIBC tissues, 91 tsRNAs were significantly differentially expressed. Then, four candidate tsRNAs, tiRNA-1:34-Val-CAC-2, tiRNA-1:33-Gly-GCC-1, tRF-1:32-Gly-GCC-1, and tRF-+1:T20-Ser-TGA-1, were selected. Next, a bioinformatics analysis showed the potential target genes and tsRNA–mRNA network. The most significant and meaningful terms of gene ontology were the positive regulation of the phosphate metabolic process, lamellipodium, and protein-cysteine S-acyltransferase activity in the biological process, cellular component, and molecular function, respectively. In addition, the top four pathways were predicted by the Kyoto Encyclopedia of Genes and Genomes database (KEGG). Finally, qRT-PCR demonstrated a similar expression pattern compared to sequencing data for the candidate tsRNAs. In short, we find differential expression profiles and predict that *tiRNA-1:33-Gly-GCC-1, tRF-1:32-Gly-GCC-1*, and *tRF-+1:T20-Ser-TGA-1* are very likely to engage in the pathophysiological process of MIBC via regulating the target genes in the key pathways.

## 1. Introduction

Bladder cancer is the sixth most prevalent cancer and the second most prevalent malignancy of the urinary system among men worldwide [1]. Bladder cancer leads to a large societal burden [2]. In 2015, the incidence of bladder cancer was 5.80/100,000, ranking 13th among all malignancies, and the mortality rate of bladder cancer was 2.37/100,000, ranking 11th in China [3]. Therefore, bladder cancer is the most common malignant tumor in urology in China. It represents a range of diseases, including non-muscle-invasive bladder cancer (NMIBC) (Ta, Tis, T1) and muscle-invasive bladder cancer (MIBC) (T2–T4), depending on the detrusor muscle invasion [4]. Urothelial bladder cancer, previously often described as transitional cell cancer, is the predominant histological type in China [5].

MIBC accounts for approximately 20% of all bladder cancer in China. Patients with MIBC usually have a poor prognosis. Based on data, approximately 50% of patients ultimately develop the disease at distant sites because of disseminated micrometastases [6]. Thus, compared to NMIBC, which is not aggressive and is usually noninvasive, MIBC is far more metastatic and life-threatening [7]. In the past ten years, increased interest in clinical treatment development have rapidly expanded the treatment armamentarium, which has reduced disease-specific mortality but is not sufficient. Hence, novel and multimodal therapies playing a key role in conjunction with local therapy should be developed and applied to reduce the rates of recurrence and mortality. Patients with MIBC present a greatly variable disease course despite similar clinicopathological traits [8]. The management of the disease relies on lifelong surveillance strategies with invasive interventions, mainly cystoscopy before surgery, which adversely affects patients’ quality of life and leads to a high economic burden on healthcare systems [9]. Thus, besides the traditionally established clinical markers, more precise diagnosis and treatment methods are needed. With increasingly more evidence of the strong molecular heterogeneity of bladder cancer [10,11], the exploration of the molecular background of bladder cancer could lead to the establishment of novel molecular markers and gene therapies to improve the clinical diagnosis and treatment for patients with bladder cancer, especially MIBC.

The majority of the human genome is noncoding RNAs (ncRNAs), which are involved in the regulation of gene expression through post-transcriptional levels and epigenetic modalities. Recently, numerous studies focused on ncRNAs, including long noncoding RNAs (lncRNAs) [12], microRNAs (miRNAs) [13], and circular RNAs (circRNAs) [14], as important contributors to the pathophysiological changes in bladder cancer, could be beneficial in that regard [15]. Transfer RNAs (tRNAs), which serve as a connector molecule involved in decoding messenger RNAs (mRNAs) and translating proteins, were the first group of ncRNAs to be characterized [16]. tRNAs are also the most abundant short ncRNAs. Furthermore, with the update and usage of next-generation sequencing methods, many researchers have found many small ncRNAs with lengths of 18–40 nts cleaved from tRNAs, called tRNA-derived small RNAs (tsRNAs) [17]. Similar to miRNAs, tRNAs undergo a specific maturation process, whereby longer primary transcripts are specifically cleaved by nucleases to produce a large number of different classes of small ncRNAs. Generally, tsRNAs can be roughly divided into two groups depending on the length and cleavage sites of the tRNAs: tRNA-derived fragments (tRFs) with 16–28 nucleotides and tRNA-derived stress-induced RNAs (tiRNAs) with 29–50 nucleotides. Numerous studies have shown that they are produced by precise biogenic processes instead of meaningless cleavages of tRNAs [18]. Dissimilar to miRNA: (i) the process of tsRNA biosynthesis is more complex; (ii) tsRNA-mRNA binding is more extensive; (iii) the structure of tsRNAs is more complex than miRNAs, and RNA modifications are more extensive (acetylation and methylation at both ends), resulting in a different detection process from miRNAs; (iv) most importantly, tsRNAs are associated with argonaute protein (AGO) types 1–4, while the main effector protein for miRNAs function is AGO2. The biogenesis of tsRNAs is highly conserved and structure-dependent, and they have been reported to play key regulatory roles in the pathophysiological processes of various diseases, for example, cancer and inherited metabolic diseases. For instance, specific tiRNAs may act as diagnostic and prognostic biomarkers in patients with clear-cell renal cell cancer [19] and prostate cancer [20]. Additionally, tRF-3001b may aggravate the development of nonalcoholic fatty liver disease by inhibiting autophagy via targeting Prkaa1 [21]. Interestingly, some sperm tsRNAs have been reported to regulate the intergenerational inheritance of diet-induced metabolic disease as epigenetic factors [22]. In addition, some studies have demonstrated multiple tsRNAs are generated under stress conditions and involved in gene expression regulation by targeting genes in a miRNA-like manner [23,24]. In terms of bladder cancer, only one study has reported increased *5′-tRF-LysCTT* levels, which were strongly negatively correlated with adverse pathological features and prognoses mainly using bioinformatics [25]. Therefore, we may rationally speculate that specific tsRNAs may be associated with pathophysiological changes in bladder cancer, especially more aggressive and invasive MIBC.

**Figure 1:**
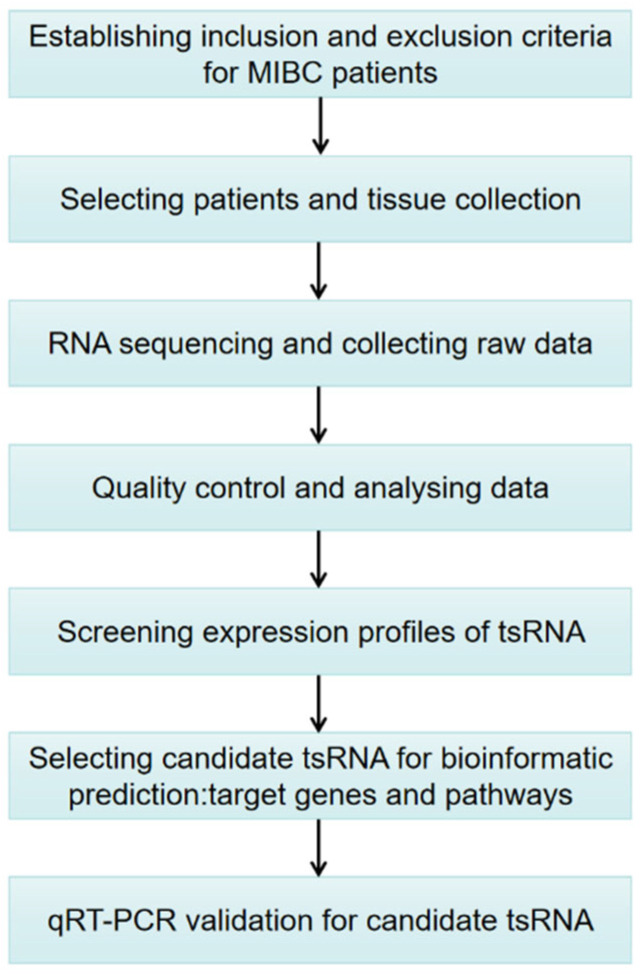
Study design illustration. MIBC, muscle-invasive bladder cancer; tsRNAs, tRNA-derived small RNA; qRT-PCR, quantitative real-time polymerase chain reaction.

To date, the expression patterns of tsRNA in MIBC have not been comprehensively analyzed. Hence, the purpose of this investigation is to explore the tsRNAs’ spectrum and preliminarily determine the potential functional roles of candidate tsRNAs in the pathophysiology of MIBC in a Chinese population. Figure 1 shows the study design. These findings may provide a new theoretical basis for uncovering the molecular mechanism of MIBC progression, which is expected to provide new treatment strategies based on tsRNAs.

## 2. Materials and Methods

### 2.1. MIBC Patients and Sample Collection

This study included tissue samples from eight MIBC patients who had undergone surgery for MIBC at the Department of Urology of Beijing Friendship Hospital affiliated with Capital Medical University (Beijing, China) in 2021. The inclusion criteria were as follows: (1) Chinese nationality and age above 18 years; (2) clinical stage T2–4a, Mx or M0 bladder cancer, pathologically confirmed using the 8th bladder cancer TNM staging system established by the International Union Against Cancer (UICC) in 2017 [26]; (3) high-grade bladder cancer, pathologically confirmed using the World Health Organization (WHO) 2004 bladder cancer grading system [27]; (4) urothelial carcinoma without other histological types; (5) no history of other malignancies, including carcinoma in situ; (6) radical cystectomy with or without lymph node dissection. Patients with confirmed distant metastasis were excluded along with those who had received any form of neoadjuvant treatment prior to surgery. This study was approved by the Ethics Committee of Beijing Friendship Hospital affiliated with Capital Medical University and was conducted in accordance with the Declaration of Helsinki ethical standards. Informed consent was obtained from all participants. Additionally, pathological features of the eight included patients are displayed in Table 1. In this study, eight pairs of MIBC specimens and adjacent control mucosal tissues (with a distance of 3 cm from the tumor) were collected during the surgery and quickly stored in liquid nitrogen to prevent RNA degradation. Among them, four pairs of tissues (tumor and paracancerous tissues) were used for tsRNA sequencing. The sample size of sequencing and polymerase chain reaction (PCR) mainly referred to these publications [28,29,30].

### 2.2. Pre-Sequencing Preparation

In brief, we extracted total RNA using TRIzol method (Invitrogen, Carlsbad, CA, USA) according to reagent instructions. Before the tsRNA sequencing, agarose gel electrophoresis and the NanoDrop ND-1000 (NanoDrop, Wilmington, DE, USA) were used to check the integrity and quantity of each RNA sample in terms of quality control.

### 2.3. Pretreatment of tsRNA and Library Preparation

The following treatments before the library preparation for the total RNA samples were performed: 3′-aminoacyl (charged) deacylation to 3′-OH for 3′ adaptor ligation, 3′-cP (2′,3′-cyclic phosphate) removal to 3′-OH for 3′ adaptor ligation, 5′-OH (hydroxyl group) phosphorylation to 5′-P for 5′-adaptor ligations, then m1A and m3C demethylation for efficient reverse transcription. Next, sequencing libraries were size-selected for the RNA biotypes to be sequenced using an automated gel cutter. The sequencing library was created by an Agilent 2100 Bioanalyzer using an Agilent DNA 1000 chip kit (Agilent, part # 5067-1504). tRNA sequences were downloaded from GtRNAdb. Only fragments that could be compared with tRNA and pre-tRNA were defined as tsRNAs to exclude the influence of other small RNAs.

### 2.4. Libraries Denaturation and tsRNA Sequencing

The libraries were denatured and diluted to a loading volume of 1.3 mL and a loading concentration of 1.8 pM. The diluted libraries were loaded onto a reagent cartridge and forwarded to sequencing, performed on an Illumina NextSeq 500 system using NextSeq 500/550 V2 kit (#FC-404-2005, Illumina, San Diego, CA, USA) in accordance with the manufacturer’s instructions; 50 running cycles of the sequencing were set.

### 2.5. Data Collection and Analysis

The raw sequencing data were selected after passing the Illumina chastity filter. The sequencing data were trimmed and filtered by the Cutadapt software. Next, the trimmed data were aligned to the mature tRNA sequences with the NovoAlign software (v2.07.11). Differentially expressed tsRNAs were determined with R package edgeR [31]. The following tsRNAs naming system was used: ID of each tsRNA started with the prefix “tRF” or “tiRNA”, which stands for “tRNA-related fragments” or “tRNA halves”, respectively. The second part included the start and end positions of the tsRNA relative to the source tRNA. The two positions were separated by a colon. The standard Sprinzl tRNA position numbering was used. If the position was located at the leader or trailer sequence of the precursor tRNA gene, the numbering was preceded by the letter “L” or “T”, respectively. The third part was the name of the tRNA from which the tsRNAs were derived. tRNA names from Genomic tRNA Database (http://gtrnadb.ucsc.edu/, accessed on 10 December 2021) or the results predicted by tRNAscan-SE (http://trna.ucsc.edu/tRNAscan-SE/, accessed on 10 December 2021) were used. If the tsRNA was derived from a mature tRNA transcript, only the isotype, anticodon, and transcript ID were included. If the tRF was derived from a pre-tRNA gene that had a unique leader or trailer sequence, the gene copy number was also included; for example, Val-AAC-1-2. In the next part, A tsRNA may have been derived from multiple tRNAs due to identical sequences in some parts of different tRNA transcripts. If this occurred, an optional component with the prefix “M” and the number of matching tRNAs was added to the tsRNA’s ID. Finally, if there were no mismatches in the alignment between the tsRNA and the source tRNA, this field was left empty. A single descriptor had the format offset: reference-base > read-base. We used the counts per million (CPMs) of the total aligned tRNA reads to measure and normalize the tsRNA expression levels. When comparing the two groups for profile differences (tumor versus paracancerous tissue), the “fold change (FC)”, i.e., the ratio of the group averages between the groups, was computed for each tsRNA. An FC > 1.5 and *p* < 0.05 defined a significantly different expression. Correlation analysis, volcano plots, pie plots, hierarchical clustering, Venn plots, scatter plots, and principal component analysis (PCA) were produced with R software.

### 2.6. Bioinformatic Prediction

First, the tsRNAs had seed sequences matching the crosslink-centered regions of the target genes [32]. A meta-analysis has shown that tsRNAs could silence the target gene through complementary base pairing [33]. Here, is the example [34], authors found that *tsRNA-16902*, a 3′-half, could regulate hMSC adipogenic differentiation by targeting RARγ via the Smad2/3 signaling pathway. Additionally, the direct action of tsRNA-16902 and RARγ was shown by luciferase reporter. For bioinformatic prediction, the target prediction integrated the following algorithms: (1) An old dynamic programming algorithm based on RNA secondary structure and free energy. Any seed type locus could be found. (2) According to the fitting of mRNA and tsRNA expression profile data, some scoring models with biological significance, site sequence characteristics, and relative conservatism were found. It could search the perfect matching sites of 8mer, 7mer-m8, and 7mer-1a nucleotides 2-6, 2-7, and 2-8. In accordance with previous studies [29,35], three common algorithms were used to predict the tsRNA targets, including TargetScan (http://www.targetscan.org, accessed on 10 January 2022), miRanda (http://www.microrna.org, accessed on 10 January 2022), and miRDB (http://www.mirdb.org, accessed on 10 January 2022). Notably, to reduce the false-positive results, only genes predicted by all three algorithms were considered target mRNAs of the tsRNAs. The tsRNA/mRNA network was visualized by Cytoscape (version 3.5.1, the Cytoscape Consortium, San Diego, CA, USA). Second, Gene Ontology (GO, http://www.geneontology.org, accessed on 20 January 2022) was used to show cellular and molecular function of the target genes. Significant pathways were predicted by the Kyoto Encyclopedia of Genes and Genomes database (KEGG, www.genome.jp/kegg, accessed on 20 January 2022). The GO and KEGG pathway terms with *p* < 0.05 and minimum count of 3 and enrichment factor of >1.5 were seen as significance. The false discovery rate (FDR) was calculated to correct the *p* values. More specifically, *p* values were calculated based on an accumulative hypergeometric distribution.

### 2.7. Quantitative Reverse Transcription-PCR

In total, eight pairs of tissues (tumor and paracancerous) were used in PCR. RNAs’ concentration and purity were assessed using NanoDrop ND-1000, and RNA integrity was verified by denatured agarose gel electrophoresis. Next, RNA pretreatment and cDNA synthesis were performed using rtStarTM tRF and tiRNA Pretreatment Kit (Cat# AS-FS-005, Arraystar, Rockville, MD, USA) and rtStarTM First-Strand cDNA Synthesis Kit (Cat# AS-FS-003, Arraystar), respectively. Quantitative reverse transcription-PCR (qRT-PCR) was performed in a ViiA 7 Real-time PCR System (Applied Biosystems, Waltham, MA, USA) using a 2× PCR master mix (Arraystar: AS-MR-006-5). The parameter settings were as follows: 95 °C denaturation (10 min), 95 °C (10 s), and 60 °C (60 s), followed by 40 cycles (fluorescent signals were measured). After the end of the amplification reaction, the procedure was performed as follows: 95 °C (10 s), 60 °C (60 s), and 95 °C (15 s). The results were analyzed with the 2^−ΔΔCT^ method. U6 was used for endogenous control gene. All reactions were performed in triplicate.

### 2.8. Statistical Analysis

SPSS software (version 21.0, Chicago, IL, USA) was used as basic software for statistical analysis. GraphPad Prism (version 8.2.1.441) was used to prepare graphs. The results were shown as the mean ± standard error of the mean (SEM). The data passed the Shapiro–Wilk (W) normality test. Student’s t test (paired, two-tailed) was used to analyze the significance of differences between the two groups. FDR was calculated from Benjamini–Hochberg FDR to correct the *p* value (q-value). *p* < 0.05 was considered significant.

### 2.9. Database and Accession Numbers

The accession number is GSE192651 for the data of the tsRNA-Seq deposited at the Gene Expression Omnibus (GEO) database.

## 3. Results

### 3.1. Altered Expression Profiles of tsRNAs in MIBC Tissues

The tsRNA-Seq analysis was used to identify the tsRNA expression levels in the two groups. First, in terms of sequencing quality control, the quality score plot of each sample was shown (shown in Supplementary Figures S1 and S2 and Supplementary Table S1). The quality score Q was logarithmically relevant to the base calling error probability (*p*). Generally, Q30 indicates the incorrect base calling probability of 0.001 or 99.9% base calling accuracy. Thus, a Q score above 30 (>99.9% correct) represented high-quality data. Based on the results of quality control, the proportion of base (Q ≥ 30) numbers was more than 93% in each sample, indicating that all samples passed the quality test.

Next, we calculated the correlation coefficients for all pairs of the samples. The results indicated that the two compared samples were similar (shown in Supplementary Figure S3A). In addition, PCA was used to explore the sample classes based on the expression. The results showed distinguishable tsRNA expression profiles among eight samples (shown in Supplementary Figure S3B). The results showed a high similarity of data within each group and a low similarity of data between the groups. In total, an altered expression of 574 tsRNAs was identified between tumor and paracancerous tissues. As shown in the Venn diagram in Supplementary Figure S3C, there were 216 commonly expressed tsRNAs, 134 tsRNAs specifically expressed in the tumor tissue, and 29 tsRNAs specifically expressed in paracancerous tissue. As shown in the Venn diagram in Supplementary Figure S3D, a total of 76 dysregulated tsRNAs in this study was known tRFs from tRFdb database, while 498 specific tsRNA were detected that had not been previously known.

**Figure 2:**
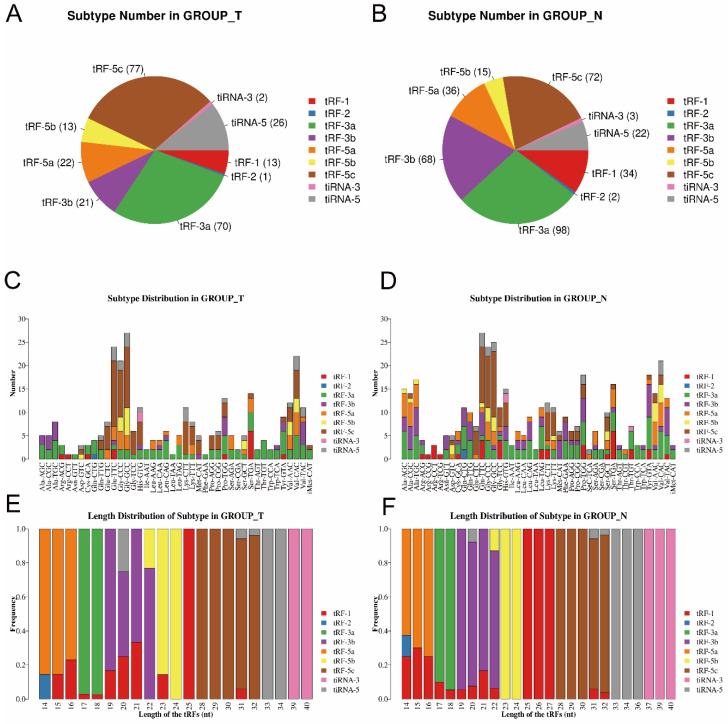
Stacked bar chart (T, tumor group. N, paracancer group). (**A**,**B**) Pie chart of the distribution of tsRNA subtypes in T group and N group. (**C**,**D**) Subtype number in T and N group. X axes: tRNA isodecoders; Y axes: the number of all subtype tsRNAs. The color: the subtype of tsRNAs. (**E**,**F**) Length distribution of subtype. The X axes: length of tsRNAs; Y axes: the frequency of the subtype; the color: the subtype of tsRNAs.

Then, as shown in Figure 2A,B, pie charts were used to illustrate each tsRNA subtype in the two groups, indicating that most of the tsRNAs detected in sequencing were derived from mature tRNAs (except for tRF-1). Among them, tRF-3a and tRF-5c accounted for the largest proportion. Compared to the paracancerous group, the proportion of tRF-5c was larger in the tumor group. Then, as shown in Figure 2C,D, the number of tsRNAs subtypes was counted against tRNA isodecoders. The stacked bars represent different tRNA isodecoders on top of each other. The height of the resulting bar indicates the combined results of tRNA isodecoders. As shown in Figure 2E,F, the height of the resulting bar showed the combined results of the length of the tsRNAs. The frequencies of the subtypes against the length of the tsRNAs were compared via the stacked bar charts.

**Figure 3:**
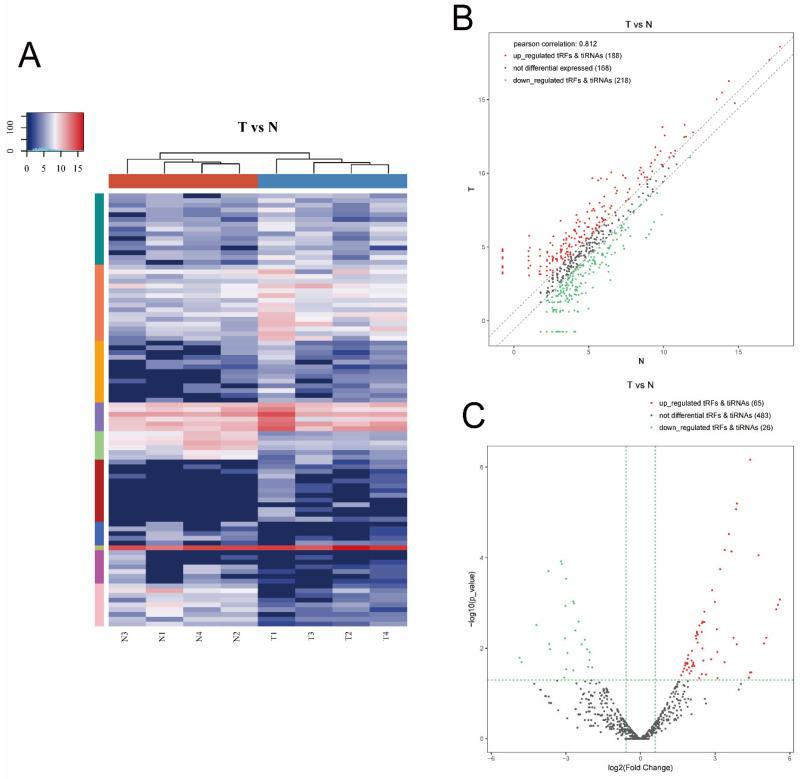
Hierarchical clustering and difference between the two groups (T vs. N). (**A**) The hierarchical clustering heat-map for the tsRNAs. The color in the panel represents the relative expression level (*log2*-transformed). Blue represents an expression level below the mean, and red represents an expression level above the mean. (**B**) The scatter plots of differentially expressed tsRNAs. tsRNAs above the top line (red dots, upregulation) or below the bottom line (green dots, downregulation) show more than 1.5-fold change between the two compared groups. Gray dots indicate nondifferentially expressed tsRNAs. (**C**) The volcano plots of significantly differentially expressed tsRNAs. The values of the X and Y axes in the volcano plot were *log2*-transformed fold change and -log10-transformed *p*-values between the two groups, respectively. Red/green circles indicate statistically significant differentially expressed tsRNAs with a FC of no less than 1.5 and a *p*-value < 0.05 (red: upregulated; green: downregulated). Gray circles indicate nondifferentially expressed tsRNA with FC and/or q-value, which did not meet the cutoff thresholds.

Hierarchical clustering was determined to visualize the differentially expressed tsRNAs. Figure 3A presents a distinguishable expression profiling of tsRNAs among tissues. Figure 3B shows scatter plots that present the tsRNA expression variation (or reproducibility) between the two groups using the FC. We found 406 tsRNAs were altered expressed between the T and N group (cutoff: FC > 1.5). Among them, 188 tsRNAs were upregulated, whereas 218 were downregulated. Moreover, a Pearson correlation coefficient of 0.812 was calculated, suggesting a relatively close relationship between the groups. As shown in Figure 3C, volcano plots were created with the FC and *p* values to visualize the significantly differential expression between the two groups. In total, 91 tsRNAs were significantly differentially expressed between the two groups (cutoff: FC > 1.5 and *p* value < 0.05). Among them, compared with paracancerous tissues, 65 tsRNAs were overexpressed, whereas 26 were underexpressed in tumor tissues. The experimental data regarding the top 10 upregulated and downregulated tsRNAs ranked by FC are shown in Table 2.

### 3.2. Target Gene Prediction

**Figure 4:**
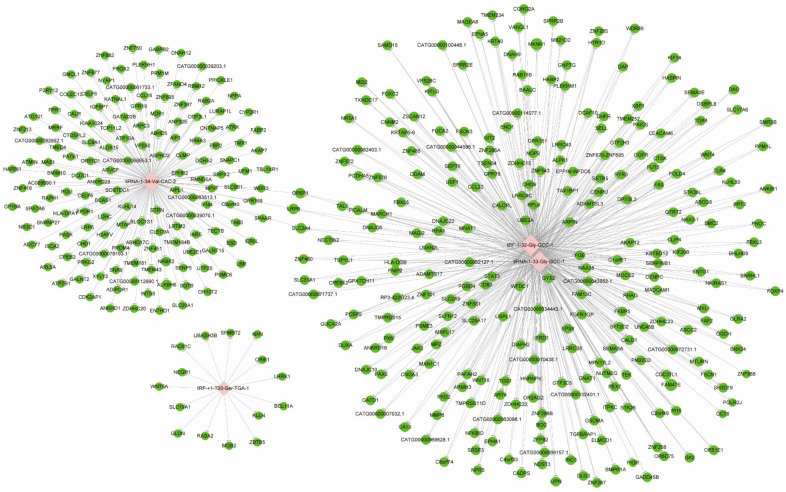
The tsRNA/mRNA network analysis. The network included the four candidate tsRNAs and their predicted target mRNAs (nodes in red color are tsRNAs; nodes in light-green color are target mRNAs).

After screening and filtering the original data, the four significantly differentially expressed tsRNAs were determined for a further analysis. The screening criteria were: (1) higher FC, lower *p* value; (2) higher CPM; (3) referring to previous publications. The four significantly differentially expressed tsRNAs, namely, *tiRNA-1:34-Val-CAC-2, tiRNA-1:33-Gly-GCC-1, tRF-1:32-Gly-GCC-1, and tRF-+1:T20-Ser-TGA-1*, were, subsequently, analyzed by bioinformatic methods. Among them, three tsRNAs were significantly upregulated, while one tsRNA was significantly downregulated in MIBC tissues (Table 2). Next, there was evidence [33] that tsRNAs could recognize mRNA targets using their seed sequence (positions 2–7 nt at their 5′-end) and inhibit the mRNA translational activities with AGO protein. Thus, tsRNA/mRNA interaction networks were visualized (Figure 4). In total, 154, 256, 256, and 15 target genes were predicted to have a close relationship with tiRNA-1:34-Val-CAC-2, tiRNA-1:33-Gly-GCC-1, tRF-1:32-Gly-GCC-1, and tRF-+1:T20-Ser-TGA-1, respectively.

### 3.3. Signal Pathways Analysis

**Figure 5:**
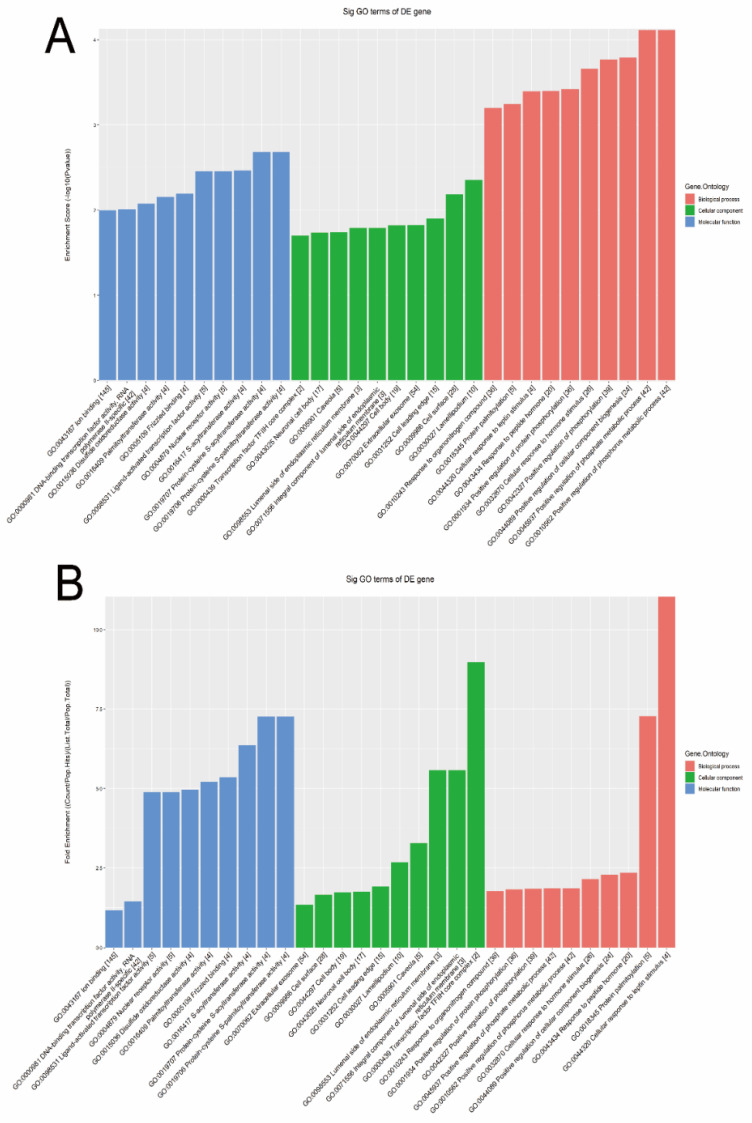
The general GO annotations enrichment score for cellular component, molecular function, and biological processes of the target mRNAs regulated by the four candidate tsRNAs. (**A**) Enrichment score. (**B**) Fold enrichment.

The GO bioinformatic analysis was performed. Next, we determined a classification for the significantly enriched terms of target genes of the four candidate tsRNAs, ranking them by a top 10 enrichment score and fold enrichment with counts and *p* values. In terms of the enrichment score (shown in Figure 5A), the results showed that the most significant and meaningful terms were the positive regulation of the phosphate metabolic process (GO:0045937), lamellipodium (GO:0030027), and protein-cysteine S-acyltransferase activity (GO:0019707) in the biological process, cellular component, and molecular function, respectively (Supplementary Figure S4). This suggested the functional roles of the target genes of the candidate tsRNAs at the tumor metabolism level. In terms of the fold enrichment (shown in Figure 5B), the results showed that the most significant and meaningful terms were cellular response to leptin stimulus (GO:0044320), transcription factor TFIIH core complex (GO:0000439), and protein-cysteine S-acyltransferase activity (GO:0019707) in the biological process, cellular component, and molecular function, respectively (Supplementary Figure S5). This suggested that the functional roles of the target genes of the candidate tsRNAs are involved in diverse pathophysiological processes in MIBC.

**Figure 6:**
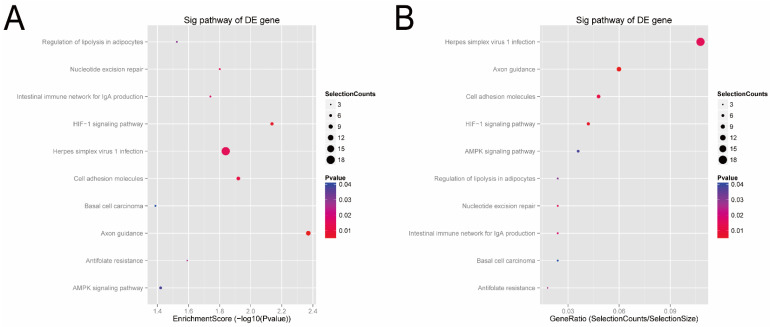
KEGG pathway analysis of the target genes of the four candidate tsRNAs. (**A**) Pathway dot plot explanation (enrichment score dot plot). The dot plot showed the top ten enrichment score (−log10(*p*-value)) value of the significant enrichment pathways. (**B**) Pathway dot plot explanation (gene ratio dot plot). The dot plot presented the gene ratio value of the top ten most significant enrichment pathways.

Next, according to the enrichment score of the KEGG analysis (Figure 6A), the top four pathways predicted were the axon guidance (hsa04360), HIF-1 signaling pathway (hsa04066), cell adhesion (hsa04514), and herpes simplex virus 1 infection (hsa05168). In terms of the gene-ratio analysis of the target genes of the candidate tsRNAs (Figure 6B), the top four pathways predicted were herpes simplex virus 1 infection (hsa05168), axon guidance (hsa04360), cell adhesion (hsa04514), and HIF-1 signaling pathway (hsa04066). In addition, all tsRNA-targeted mRNAs in these four pathways were presented in pathway maps (Supplementary Figure S6). Previous studies have reported that these pathways, especially cell adhesion (hsa04514) and HI F-1, are closely related to bladder cancer. Thus, we speculated that these four candidate tsRNAs might be involved in the pathophysiological process via these signaling pathways.

### 3.4. Verification by qRT-PCR

**Figure 7:**
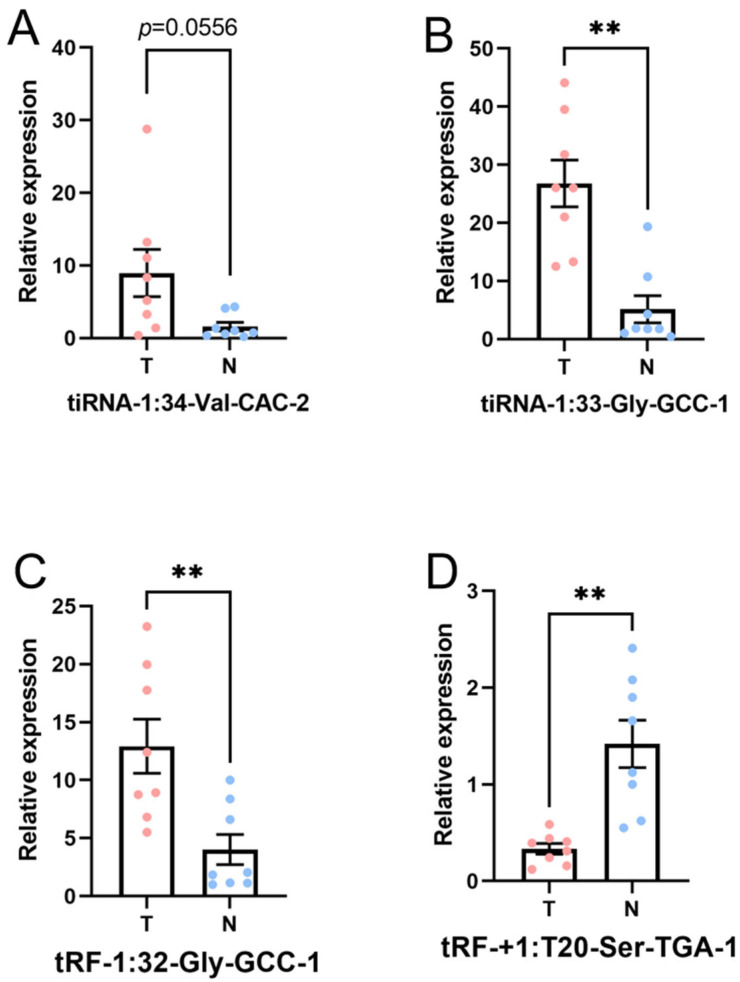
Expression level of the candidate tsRNAs (T, tumor group. N, paracancer group). The data were normalized using the mean ± SEM. (**A**) tiRNA-1:34-Val-CAC-2 (*p*-value = 0.0556), (**B**) tiRNA-1:33-Gly-GCC-1 (*p*-value < 0.01), (**C**) tRF-1:32-Gly-GCC-1 (*p*-value < 0.01), and (**D**) tRF-+1:T20-Ser-TGA-1 (*p*-value < 0.01). ** indicates *p*-value < 0.01.

In this part, the four candidate tsRNAs selected from the sequencing were validated by low-throughput qRT-PCR. Compared with paracancerous tissue, in tumor tissue, *tiRNA-1:33-Gly-GCC-1* (*p*-value < 0.01) and *tRF-1:32-Gly-GCC-1* (*p*-value < 0.01) were significantly upregulated, and *tRF-+1:T20-Ser-TGA-1* (*p*-value < 0.01) was significantly downregulated, while *tiRNA-1:34-Val-CAC-2* (*p*-value = 0.0556) was overexpressed without reaching statistical significance. Hence, there was a similar expression pattern in the sequencing and PCR data, as shown in Figure 7. The FC and *p* values of each candidate tsRNA between the groups in terms of the sequencing and PCR analysis are presented in Table 3. The information regarding the primers used in qRT-PCR is displayed in Supplementary Table S2. These PCR results together with tsRNA sequencing data verified *tiRNA-1:33-Gly-GCC-1, tRF-1:32-Gly-GCC-1*, and *tRF-+1:T20-Ser-TGA-1* at different levels.

## 4. Discussion

Here, we revealed the expression profiles of the tsRNAs in MIBC in a Chinese population with the help of next-generation sequencing. Then, the bioinformatics suggested four tsRNAs might be involved in the regulation of signal pathways, such as axon guidance, HIF-1 signaling pathway, cell adhesion, and herpes simplex virus 1 infection. Next, the expressions of the four candidate tsRNAs were tested by PCR, and three of them were verified as significant. Taken together, the results showed these candidate tsRNAs were closely related to the pathophysiological processes of MIBC. The present study offers the first comprehensive presentation of tsRNA expression profiles and a subsequent bioinformatics analysis in MIBC, thereby providing great possibilities for future research.

In 1977 [36], tsRNA was initially detected in the urine of patients with cancer. Interestingly, tsRNAs have originally been considered the byproducts of random tRNA cleavage. It was not until 2009 that the mechanism of tsRNA production was initially revealed. Some researchers [37] found in mammalian cell lines (HepG2, HeLa, and HEK293) that angiogenin can be induced by a variety of stress conditions to cleave the anticodon rings of tRNAs to produce tsRNAs. With the discovery of tsRNA production mechanisms, tsRNAs have gradually emerged as a key molecular in multiple regulatory processes. For instance, tsRNAs can regulate mRNA stability, translation, rRNA synthesis, and RNA reverse transcription, and then regulate cell apoptosis and cell cycle; hence, they play biological roles in the occurrence of diseases [37].

Mounting evidence shows the critical function of tsRNAs as regulatory factors in tumors. For instance, recently, a 3-tsRNA has been stated to enhance cell proliferation, migration, and invasion in gastric cancer by targeting *FBXO47* [38]. *tDR-0009* induced by hypoxia could be involved in the chemoresistance of triple-negative breast cancer via the regulation of the activation of the phosphorylation of STAT3 [39]. Moreover, the expression of tsRNA is tissue-specific and spatiotemporal especially, and many studies of tsRNAs as clinical markers have emerged. In patients with clear-cell renal cell carcinoma, *5’-tRNA-Arg-CCT, 5’-tRNA-Glu-CTC*, and *5’-tRNA-Lys-TTT* halves might act as noninvasive biomarkers [19]. Moreover, androgen-dependent tsRNAs (*5’-tRNA-Glu-CUC*) can potentially act as biomarkers to monitor and predict the progression of prostate cancer [20]. Moreover, some specific tsRNAs may be promising and effective candidates as highly sensitive, noninvasive biomarkers for pancreatic ductal adenocarcinoma [40]. Recent studies about the functions and mechanisms of tsRNAs in tumors have mainly focused on regulating the target mRNA stability similar to miRNAs, namely, inhibiting target genes with the combination of AGO2 protein [33]. However, there is some evidence that the specific regulatory mechanisms of tsRNAs are not exactly the same as those of miRNAs. Goodarzi et al. [41] discovered that tsRNAs from *tRNAGlu, tRNAAsp, tRNAGly*, and *tRNATyr* could inhibit the stability of multiple proto-oncogene transcripts in breast cancer cells by competitively binding to the RNA-binding protein YBX1. *5’-tRFGln* was able to inhibit translation without target sequences complementary to mRNA, and its mechanism of action relied on conserved residues “GG” [42]. Thus, the functional roles and mechanisms of tsRNAs in cancer need to be further explored.

In our study, results of the sequencing indicated that the expression levels of tsRNAs were significantly altered in MIBC tissues compared to the adjacent mucosa. Based on the similar function of canonical miRNAs, potential target-binding mRNAs for the four candidate tsRNA were determined and filtered to construct tsRNA/target gene interactions using the bioinformatic analysis. For the pathway analysis, the most significant and meaningful terms were the positive regulation of the phosphate metabolic process [43], lamellipodium [44], and protein-cysteine S-acyltransferase activity [45], which are closely related to the occurrence and progression of bladder cancer. Moreover, some researchers have explored the relationships between bladder cancer and the four pathways identified in our study, including the axon guidance [46], HIF-1 signaling pathway [47], cell adhesion [48], and herpes simplex virus 1 infection [49]. Finally, using PCR, we validated that *tiRNA-1:33-Gly-GCC-1, tRF-1:32-Gly-GCC-1*, and *tRF-+1:T20-Ser-TGA-1* were significantly altered, showing the consistent trend to the sequencing data, which indicated that these three candidate tsRNAs might be involved in the pathophysiological process of MIBC via regulating the target genes in the key pathways.

However, this study had several limitations. First, this study only examined the expression profile and bioinformatics prediction; thus, identifying the functions of the candidate tsRNAs in vitro and in vivo is necessary in the future. Next, future work should also focus on the expression and functions of tsRNAs in NMIBC. Last but not least, the sample number in our study limited the exploration of clinical markers of tsRNAs. More studies should focus on the validation of clinical markers of specific tsRNAs in MIBC.

## 5. Conclusions

In summary, we found differential expression profiles and predicted that *tiRNA-1:33-Gly-GCC-1, tRF-1:32-Gly-GCC-1*, and *tRF-+1:T20-Ser-TGA-1* might be involved in the pathophysiological process of MIBC via regulating the target genes in the key pathways. This study provides novel insights for future investigations to explore the mechanisms and therapeutic targets for MIBC.

## Figures and Tables

**Table 1:** Patient and tumor characteristics.

Number	Age (Year)	Sex	TNM Stage and Grade	Tumor Size and Pathological Description
1	65	Female	T3aN2M0 High grade	Multifocal tumor with the biggest size of 3.0 cm × 2.5 cm located in the left front bladder wall; urothelium carcinoma with necrosis and 3/4 lymph node metastasis beside the left iliac blood vessels
2	64	Male	T3bN1M0 High grade	Multifocal tumor with the biggest size of 8.0 cm × 4.3 cm located in the posterior bladder wall; urothelium carcinoma with neurovascular invasion and 1/3 lymph node metastasis beside the right iliac blood vessels
3	80	Male	T3aN0M0 High grade	Multifocal tumor with the biggest size of 4.0 cm × 3.3 cm located in the right bladder wall; urothelium carcinoma without neurovascular invasion and node metastasis
4	63	Male	T3aN0Mx High grade	Multifocal tumor with the biggest size of 3.4 cm × 2.7 cm located in the left bladder wall; urothelium carcinoma with blood vessel invasion and without node metastasis
5	78	Male	T2aN2Mx High grade	Unifocal tumor with the size of 3.8 cm × 2.6 cm located in the left front bladder wall; urothelium carcinoma with lymphatic vessel invasion and with 2/3 lymph node metastasis beside the left iliac blood vessels
6	77	Male	T2aN0Mx High grade	Unifocal tumor with the size of 3.0 cm × 1.8 cm located in the right bladder wall; urothelium carcinoma with vascular tumor thrombus and without lymph node metastasis
7	65	Male	T3aN0Mx High grade	Multifocal tumor with the biggest size of 1.8 cm × 1.6 cm located in the posterior wall; urothelium carcinoma with vascular tumor thrombus and without lymph node metastasis
8	61	Male	T2aN0Mx High grade	Multifocal tumor with the biggest size of 1.3 cm × 1.2 cm located in the right wall; urothelium carcinoma with vascular tumor thrombus and without lymph node metastasis

**Table 2:** The top 10 upregulated and downregulated tsRNAs ranked by fold changes (T vs. N).

Gene Name	Type	Length	Fold Change	*p*-Value	q-Value	Regulation
*tRF-+1:T23-chrM.Glu-TTC*	tRF-1	23	48.724	8.305 × 10^−4^	0.034	Up
*tRF-1:29-Gln-TTG-1-M3*	tRF-5c	29	46.138	1.093 × 10^−3^	0.035	Up
*tRF-1:28-Pro-AGG-1-M6*	tRF-5c	28	44.035	1.372 × 10^−3^	0.039	Up
*tRF-+1:T33-chrM.Phe-GAA-M1-31:C>T*	tRF-1	33	33.304	5.826 × 10^−3^	0.090	Up
*tRF-1:24-His-GTG-1*	tRF-5b	24	31.182	7.829 × 10^−3^	0.104	Up
*tiRNA-1:34-Val-AAC-1-M3*	tiRNA-5	34	26.866	8.724 × 10^−5^	0.007	Up
*tRF-1:24-Val-CAC-2*	tRF-5b	24	21.919	3.389 × 10^−2^	0.240	Up
*tRF-1:29-Glu-TTC-1*	tRF-5c	29	21.344	6.822 × 10^−7^	0.000	Up
*tiRNA-1:34-Val-AAC-2*	tiRNA-5	34	21.203	3.434 × 10^−2^	0.240	Up
*tRF-1:28-Val-CAC-2*	tRF-5c	28	20.604	4.441 × 10^−2^	0.287	Up
*tRF-+1:T23-Thr-AGT-2-2*	tRF-1	23	0.034	1.627 × 10^−2^	0.173	Down
*tRF-+1:T21-Leu-TAG-3*	tRF-1	21	0.036	2.019 × 10^−2^	0.186	Down
*tRF-+1:T15-Gly-TCC-1*	tRF-1	15	0.055	3.062 × 10^−3^	0.066	Down
*tRF-1:15-Thr-CGT-5*	tRF-5a	15	0.077	1.966 × 10^−4^	0.010	Down
*tRF-1:14-Asp-GTC-1-M3*	tRF-5a	14	0.079	8.031 × 10^−3^	0.104	Down
*tRF-+1:T18-Ile-AAT-2*	tRF-1	18	0.080	1.040 × 10^−2^	0.125	Down
*tRF-54:75-His-GTG-1-M2*	tRF-3b	22	0.109	1.190 × 10^−4^	0.009	Down
*tRF-+1:T17-Ser-TGA-1*	tRF-1	17	0.112	1.371 × 10^−4^	0.009	Down
*tRF-52:69-chrM.Tyr-GTA*	tRF-3a	18	0.119	4.422 × 10^−2^	0.287	Down
*tRF-+1:T15-Lys-CTT-2-2*	tRF-1	15	0.123	5.960 × 10^−3^	0.090	Down
The candidate tsRNAs selected for bioinformatics and PCR						
*tiRNA-1:34-Val-CAC-2*	tiRNA-5	34	14.342	8.535 × 10^−6^	0.002	Up
*tiRNA-1:33-Gly-GCC-1*	tiRNA-5	33	9.222	1.774 × 10^−4^	0.010	Up
*tRF-1:32-Gly-GCC-1*	tRF-5c	32	3.703	2.204 × 10^−2^	0.189	Up
*tRF-+1:T20-Ser-TGA-1*	tRF-1	20	0.154	9.193 × 10^−4^	0.034	Down

**Table 3:** Comparison for candidate tsRNAs expression in sequencing and PCR (T vs. N; FC, fold change.).

Gene Name	Sequencing			PCR		
	FC	*p*-Value	Regulation	FC	*p*-Value	Regulation
*tiRNA-1:34-Val-CAC-2*	14.342	8.535 × 10^−6^	Up	7.925	0.0556	Up
*tRF-1:32-Gly-GCC-1*	3.703	2.204 × 10^−2^	Up	4.349	0.0082	Up
*tRF-+1:T20-Ser-TGA-1*	0.154	9.193 × 10^−4^	Down	0.231	0.0014	Down
*tiRNA-1:33-Gly-GCC-1*	9.222	1.774 × 10^−6^	Up	5.869	0.0028	Up

## Data Availability

The data presented in this study are available on request from the corresponding author. The data are not publicly available due to institutional policy.

## Supplementary Materials

The following are available online at https://www.mdpi.com/article/10.3390/genes13040601/s1, Figure S1: tsRNA-seq quality score plot for T group (tumor tissue). Figure S2: tsRNA-seq quality score plot for N group (paracancerous tissue). Figure S3: the expression level analysis. Figure S4: the top-ten significant and meaningful terms of enrichment score of target genes ranked by counts and *p*-values. Figure S5: the top-ten significant and meaningful terms of fold enrichment of target genes ranked by counts and *p*-values. Figure S6: the top four pathways predicted by KEGG with all tsRNA-targeted genes shown in pathway maps. Table S1: quality score for each sample. Table S2: sequences of primers used for qRT-PCR.

## Abbreviations

MIBC: muscle-invasive bladder cancer; NMIBC, non-muscle-invasive bladder cancer; ncRNAs, noncoding RNAs; lncRNAs, long noncoding RNAs; miRNAs, microRNAs; circRNAs, circular RNAs; tRNAs, transfer RNAs; tsRNA, tRNA-derived small RNA; mRNAs, messenger RNAs; tRFs, tRNA-derived fragments; tiRNAs, tRNA-derived stress-induced RNAs; rRNA, ribosomal RNA; siRNA, small interfering RNA; qRT-PCR, quantitative reverse transcription polymerase chain reaction; FC, fold change; CPMs, counts per million; SEM, standard error of the mean; GO, Gene Ontology; KEGG, Kyoto Encyclopedia of Genes and Genomes database. GEO, Gene Expression Omnibus. UICC, the International Union Against Cancer.
