# Supplementary material for: Differential Expression Profiles and Bioinformatics Analysis of tRNA-Derived Small RNAs in Muscle-Invasive Bladder Cancer in a Chinese Population
Source: Genes (Basel). 2022 Mar 28;13(4):601. doi: 10.3390/genes13040601 (PMC9030102; doi:10.3390/genes13040601)
Supplement: Supplementary file 1 [file genes-13-00601-s001.zip › genes-1631153-supplementary.pdf]

Supplementary Material

Differential Expression Profiles and Bioinformatics Analysis of tRNA-derived Small RNAs in Muscle-Invasive Bladder Cancer in Chinese Population

Chuan Qin, Zheng-Hao Chen, Rui Cao, Ming-Jun Shi and Ye Tian *

Department of Urology, Beijing Friendship Hospital, Capital Medical University, Beijing 100068, China;
qinchuanlove@126.com (C.Q.); chenzhmed@163.com (Z.-H.C.); caorui@whu.edu.cn (R.C.); shimingjun1127@126.com (M.-J.S.)
* Correspondence: youyitianye@126.com; Tel.: +86-010-63138377

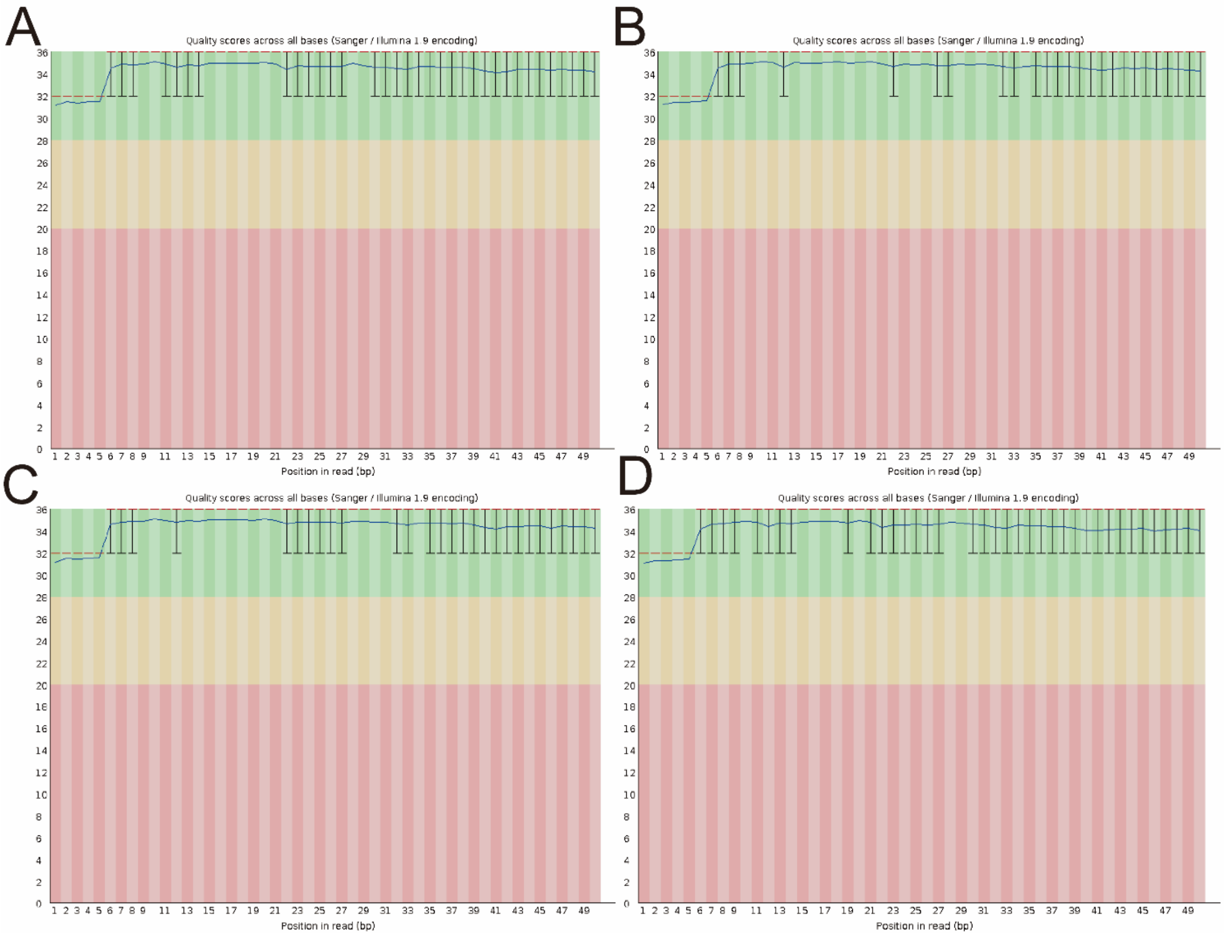

Supplementary Figure S1. tsRNA-seq quality score plot for T group (tumor tissue). The position in the read is plotted on the X-axis and the *Q* value is plotted on the Y-axis. The red line is the median *Q* score, and the blue line is the mean *Q* score. The boxplot represents the inter-quartile range, while the whiskers represent the 10% and 90% points. A Q score above 30 (>99.9% correct) is considered high quality of data. **A-D** represents tumor sample 1-4 (T1-T4).

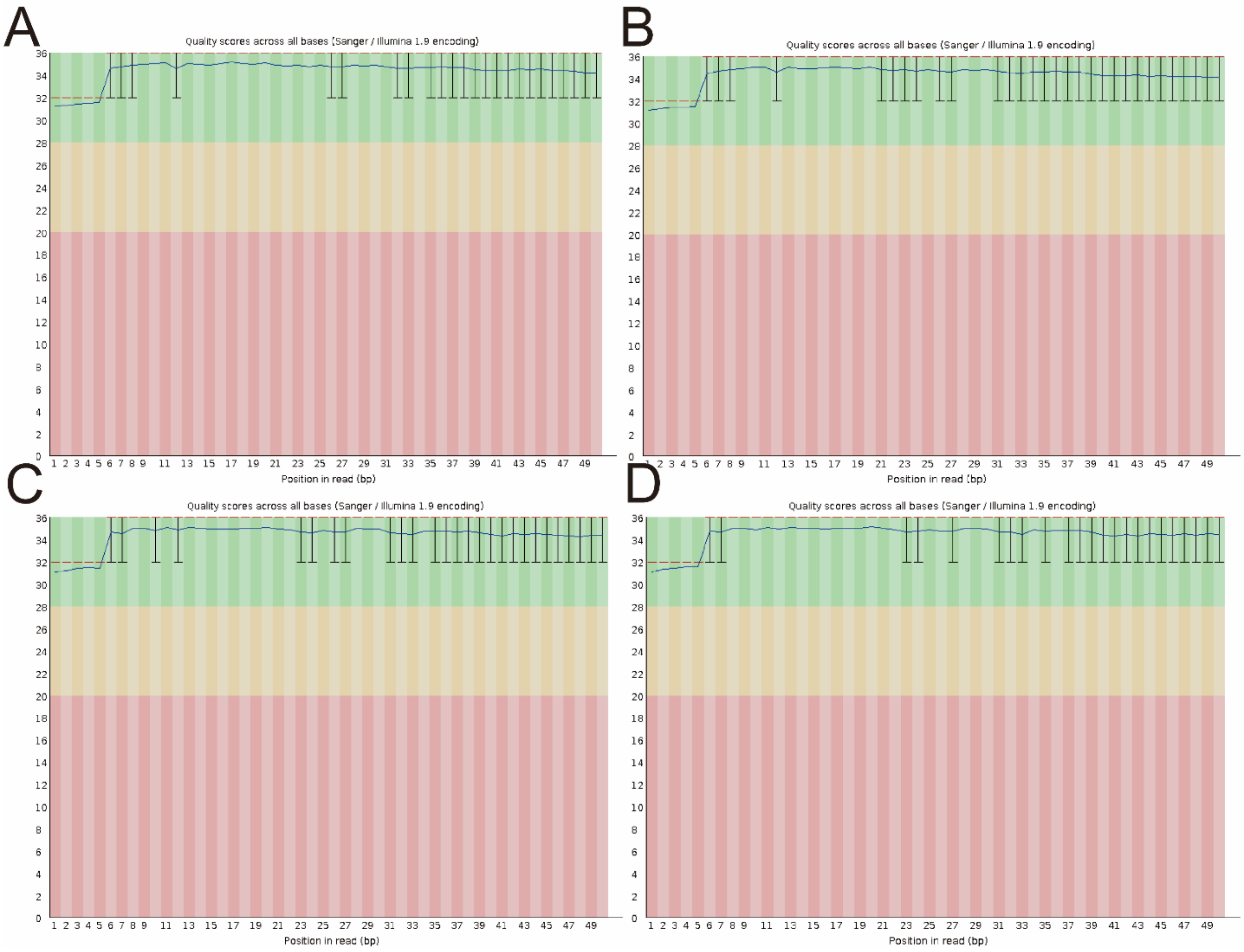

Supplementary Figure S2. tsRNA-seq quality score plot for N group (paracancerous tissue). The position in the read is plotted on the X-axis and the *Q* value is plotted on the Y-axis. The red line is the median *Q* score, and the blue line is the mean *Q* score. The boxplot represents the inter-quartile range, while the whiskers represent the 10% and 90% points. A *Q* score above 30 (>99.9% correct) is considered high quality of data. **A-D** represents paracancerous tissue 1-4 (N1-N4).

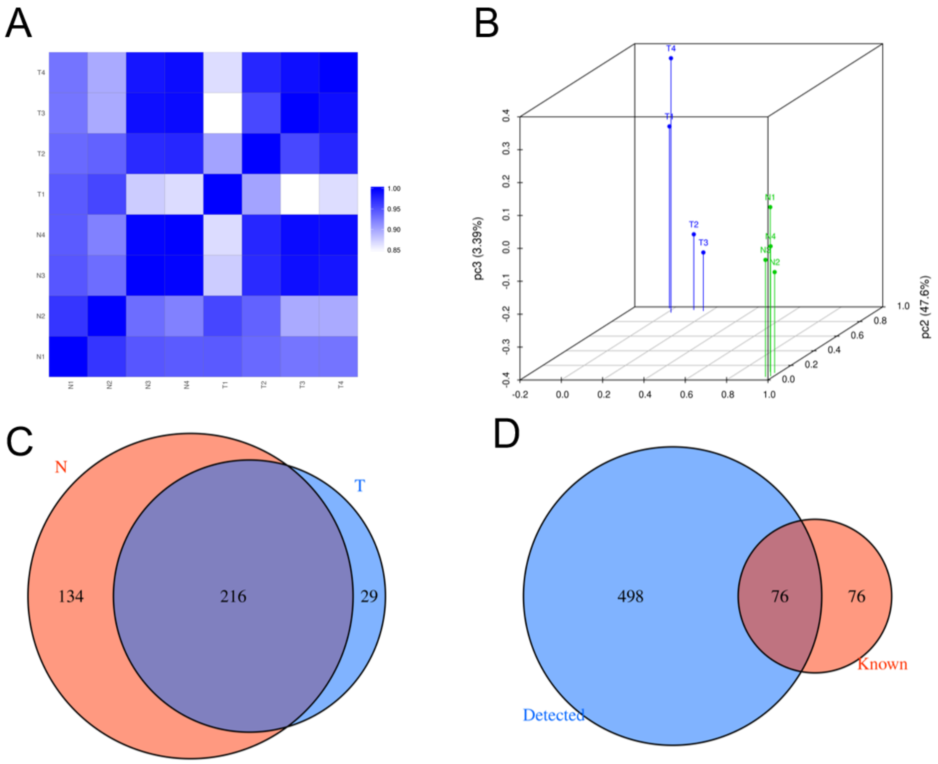

Supplementary Figure S3. The expression level analysis. (A) Heat-map of the correlation coefficient from all samples. The color in the panel represents the correlation coefficient of the two samples. The blue represents the two samples with a high correlation coefficient, and the white represents the low similarity of the two samples. **(B)** Primary component analysis: the X, Y and Z axes represent the three main factors which affected the expression level of the sample. The colored point represents the corresponding sample, and its location shows the main character of the sample. The space distance represents the similarity of the data size. **(C)** Venn diagram based on the number of commonly expressed and specifically expressed tsRNAs. This diagram shows the number of tsRNAs which were expressed in both groups and also indicated the number of the specifically expressed tsRNAs. **(D)** Venn diagram based on the number of tsRNAs known and detected. This diagram shows the number of tsRNAs detected in this study and collected in tRFdb database.

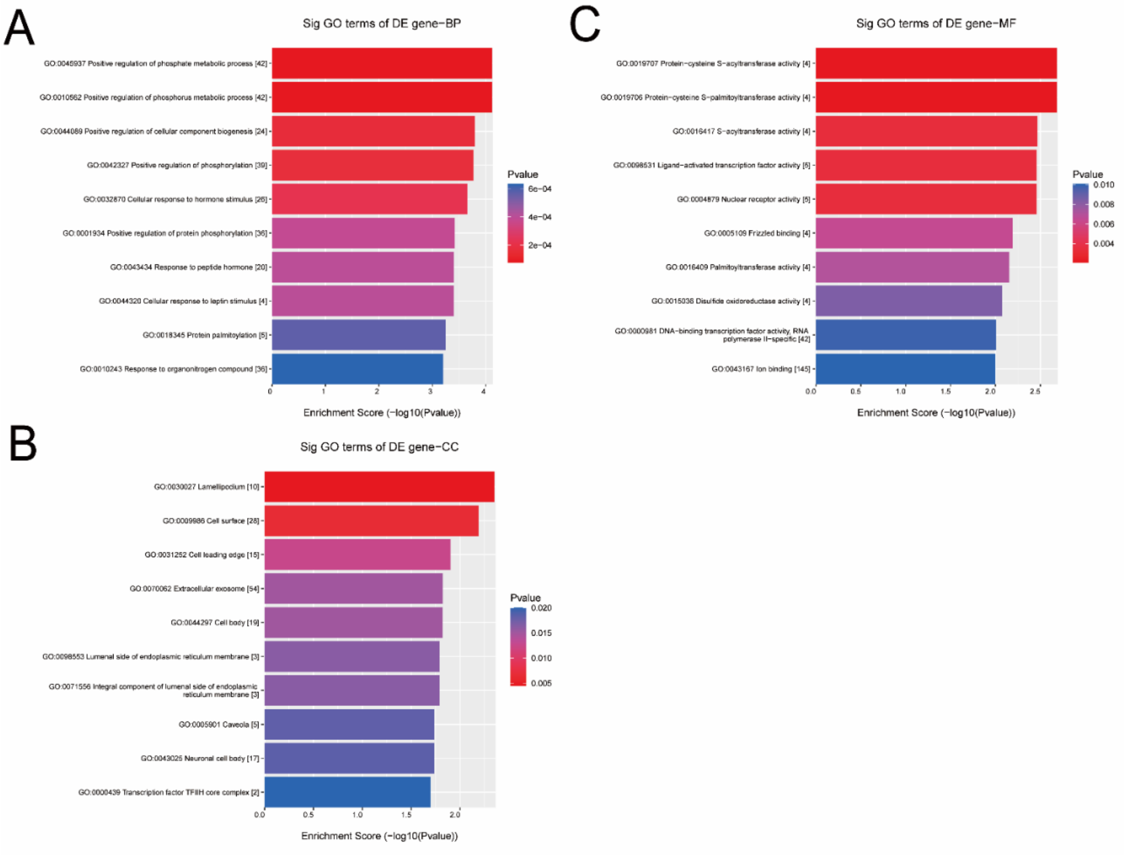

Supplementary Figure S4. The top-ten significant and meaningful terms of enrichment score of target genes ranked by counts and *P*-values. (A) biological process. (B) cellular component. (C) molecular function.

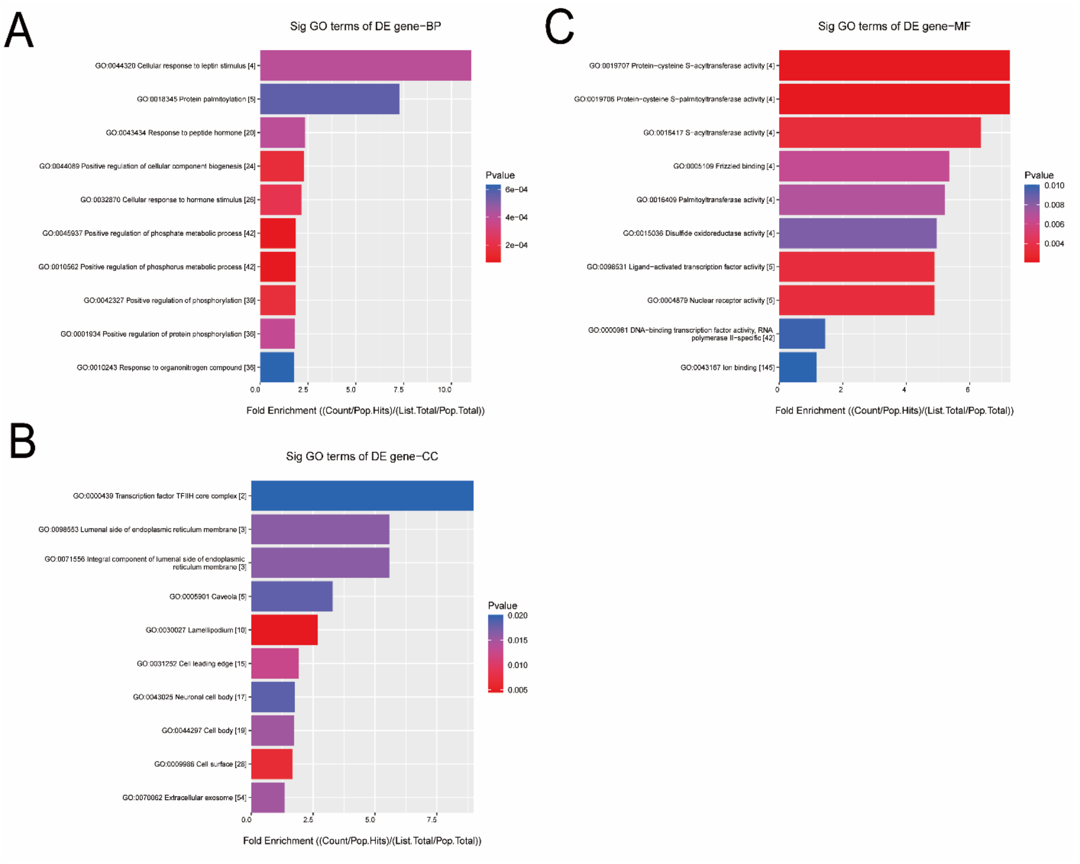

Supplementary Figure S5. The top-ten significant and meaningful terms of fold enrichment of target genes ranked by counts and *P*-values. (A) biological process. (B) cellular component. (C) molecular function

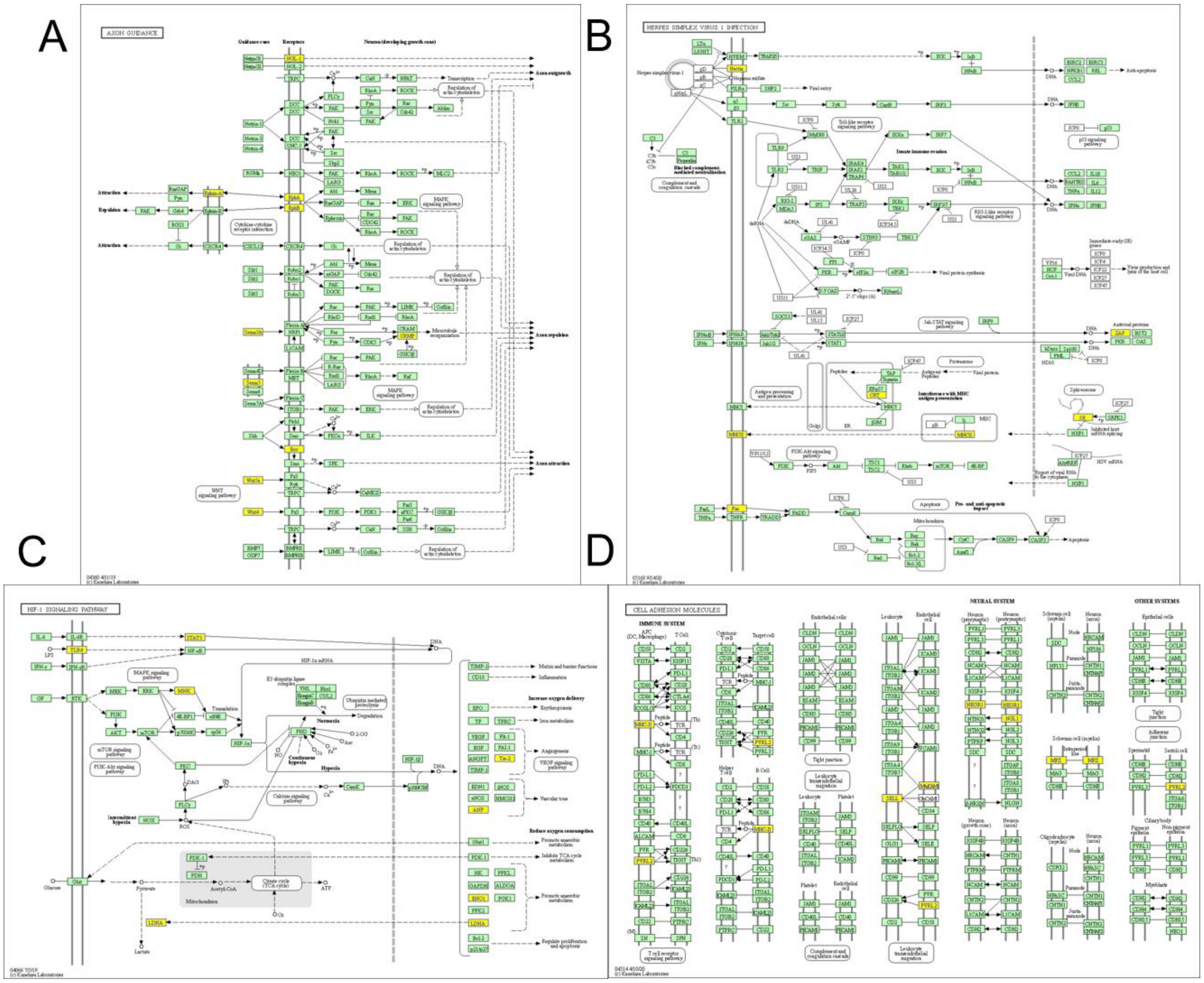

Supplementary Figure S6. The top four pathways predicted by KEGG with all tsRNA-targeted genes shown in pathway maps. Yellow marked nodes were associated with down-regulated genes, orange marked nodes were associated with up-regulated or only whole dataset genes, green nodes were not significant. (A) axon guidance (hsa04360). (B) herpes simplex virus 1 infection (hsa05168). (C) HIF-1 signaling pathway (hsa04066). (D) cell adhesion (hsa04514).

Supplementary Table S1. Quality score for each sample

Sample	TotalRead	TotalBase	BaseQ30	BaseQ30 (%)
T1	8274992	413749600	391179830	94.55
T2	7617447	380872350	361491357	94.91
T3	6159841	307992050	292418503	94.94
T4	9513040	475652000	446833685	93.94
N1	8946064	447303200	424246058	94.85
N2	8172930	408646500	386027201	94.46
N3	8346674	417333700	396028789	94.89
N4	9856730	492836500	468729835	95.11

Sample: Sample name

TotalRead: Raw sequencing reads after quality filtering

TotalBase: Number of bases after quality filtering

BaseQ30: Number of bases of Q score more than 30 after quality filtering

BaseQ30 (%): The proportion of bases (Q⩾30) number after quality filtering

Supplementary Table S2. Sequences of primers used for qRT-PCR

Gene name	Primer sequence	Ta Opt (°C)	Product size(bp)
U6	F:5’GCTTCGGCAGCACATATACTAAAAT 3’<br>R:5’CGCTTCACGAATTTGCGTGTCAT 3’	60	89
tiRNA-1:33-Gly-GCC-1	F:5’ TACAGTCCGACGATCGCATG 3’<br>R:5’ CGTGTGCTCTTCCGATCTCA 3’	60	66
tiRNA-1:34-Val-CAC-2	F:5’ GATCGCTTCTGTAGTGTAGTGG 3’<br>R:5’ CGATCTGAGGCGAACGTGA 3’	60	44
tRF-1:32-Gly-GCC-1	F:5’ TACAGTCCGACGATCGCATG 3’<br>R:5’ TCTAGGCGAGAATTCTACCACTGA 3’	60	50
tRF-+1:T20-Ser-TGA-1	F:5’ TACAGTCCGACGATCGAAGC 3’<br>R:5’ TTCCGATCTAAAATAAGAGCACC 3’	60	44
